# Rosacea pathogenesis and therapeutics: current treatments and a look at future targets

**DOI:** 10.3389/fmed.2023.1292722

**Published:** 2023-12-13

**Authors:** Garrett W. Fisher, Jeffrey B. Travers, Craig A. Rohan

**Affiliations:** ^1^Departments of Pharmacology and Toxicology, Boonshoft School of Medicine at Wright State University, Dayton, OH, United States; ^2^Dermatology, Boonshoft School of Medicine at Wright State University, Dayton, OH, United States; ^3^Department of Medicine (Dermatology), Dayton Veterans Administration Medical Center, Dayton, OH, United States

**Keywords:** rosacea, pathogenesis, inflammatory, cathelicidin, pharmacotherapeutics

## Abstract

Rosacea is a chronic inflammatory skin condition associated with a significant health and economic burden from costs and loss of productivity due to seeking medical treatment. The disease encompasses multiple phenotypic manifestations involving a complex and multi-variate pathogenesis. Although the pathophysiology of rosacea is not completely understood, ongoing research is continually elucidating its mechanisms. In this review, current concepts of rosacea pathogenesis will be addressed which involve skin barrier and permeability dysfunction, the innate and adaptive immune systems, and the neurovascular system. More specifically, the cathelicidin pathway, transient potential receptor channels, mast cells, and the NLRP3 inflammasome pathway are various targets of current pharmacologic regimens. Future therapies may seek different mechanisms to act on current treatment targets, like the potential use of JAK/STAT inhibitors in ameliorating skin barrier dysfunction or TLR antagonists in alleviating cathelicidin mediated inflammation. Other potential treatments aim for entirely different molecular targets such as microvesicle particle mediated local and systemic inflammation. Ultimately rosacea is associated with a significant health and economic burden which warrants deeper research into its pathogenesis and resultant new treatment discovery.

## 1 Introduction

Rosacea is a chronic skin condition that typically effects skin on the central face with a relatively unknown etiology and pathophysiology. Caucasians with fair skin and Fitzpatrick skin types between I and II, and those with photosensitive skin types appear to be at the greatest risk for rosacea (1). In a 2018 study, the prevalence of rosacea was estimated at 5.46% of the general population and 2.39% of all dermatologic outpatients (2). The condition is most prevalent in those above the age of 65 and is associated with increased risk of various comorbidities, which highlights the importance of diagnostic and therapeutic processes (3). Broad categories of comorbidities include cardiovascular, gastrointestinal, neurologic, psychiatric, autoimmune, and even certain malignancies (4). In general, skin diseases cause a significant global burden, and the costs and prevalence are comparable to other diseases with significant public health concerns. In 2013, rosacea was associated with a $243 million burden in treatment costs and loss of productivity in the 1.6 million people seeking medical treatment (5).

Importantly, the prevalence of rosacea in skin of color is not well understood and may be underestimated (6). Current reports of rosacea in patients of color may point to a large proportion of undiagnosed cases warranting increased awareness in populations with higher Fitzpatrick skin types (IV, V, and VI) (7). Rosacea is a clinical diagnoses, and certain visual characteristics, such as erythema, are difficult to ascertain in skin types ≥4 (8). Ultimately, the prevalence of rosacea in skin of color is likely greater than previously thought. Greater clinical diagnostic criteria and a more thorough symptom history are needed to properly diagnose rosacea in patients with darker skin types.

Rosacea is a single disease with multiple phenotypic manifestations that are important in the determination of therapeutics (9). A greater comprehension of the mechanisms of rosacea is needed to optimize future treatment options. The disease pathogenesis, although unclear, is continually elucidated with ongoing research. A multivariate set of pathogenic pathways are known, including defects in innate and adaptive immune systems, mast cells, and the neurovascular system. A hallmark of rosacea pathogenesis is its association with skin barrier and permeability dysfunction, including changes in skin hydration, pH, microbiome, and various components of the skin’s molecular structure. Many different stimuli are associated with pathogenesis from both endogenous and exogenous sources (10). The main phenotypic types of rosacea include erythematotelangiectatic (ETR), papulopustular (PPR), ocular, and acne rosacea. Vascular manifestations seen in ETR arise from neurovascular dysregulation, increased LL-37, and serine proteases (11). Papules seen in PPR and actinic rosacea are characterized by increased Th1 and Th17 cells, plasma cells, mast cells, and macrophages. Pustules seen in PPR are characterized by increased production of neutrophil-recruiting chemokines (12). Overall, studies indicate a marked upregulation of mast cell density as a common factor in all major presentations which act through innate immune responses, neurogenetic inflammation, angiogenesis, and fibrosis (13). The pathogenic processes implicated in rosacea phenotypes are discussed in greater detail in the body of this review. Current research is continually elucidating new details about the disease and new data may indicate effective therapeutic targets. For example, one Janus Kinase- signal transducer and activator of transcription (JAK/STAT) proteins, STAT3, has been shown to have significant upregulation in expression in rosacea patients compared to healthy controls (14, 15). Overall, as research into pathogenesis and drug discovery continues, we may generate new effective therapies for all rosacea phenotypes.

## 2 Disease pathogenesis

**Figure 1:**
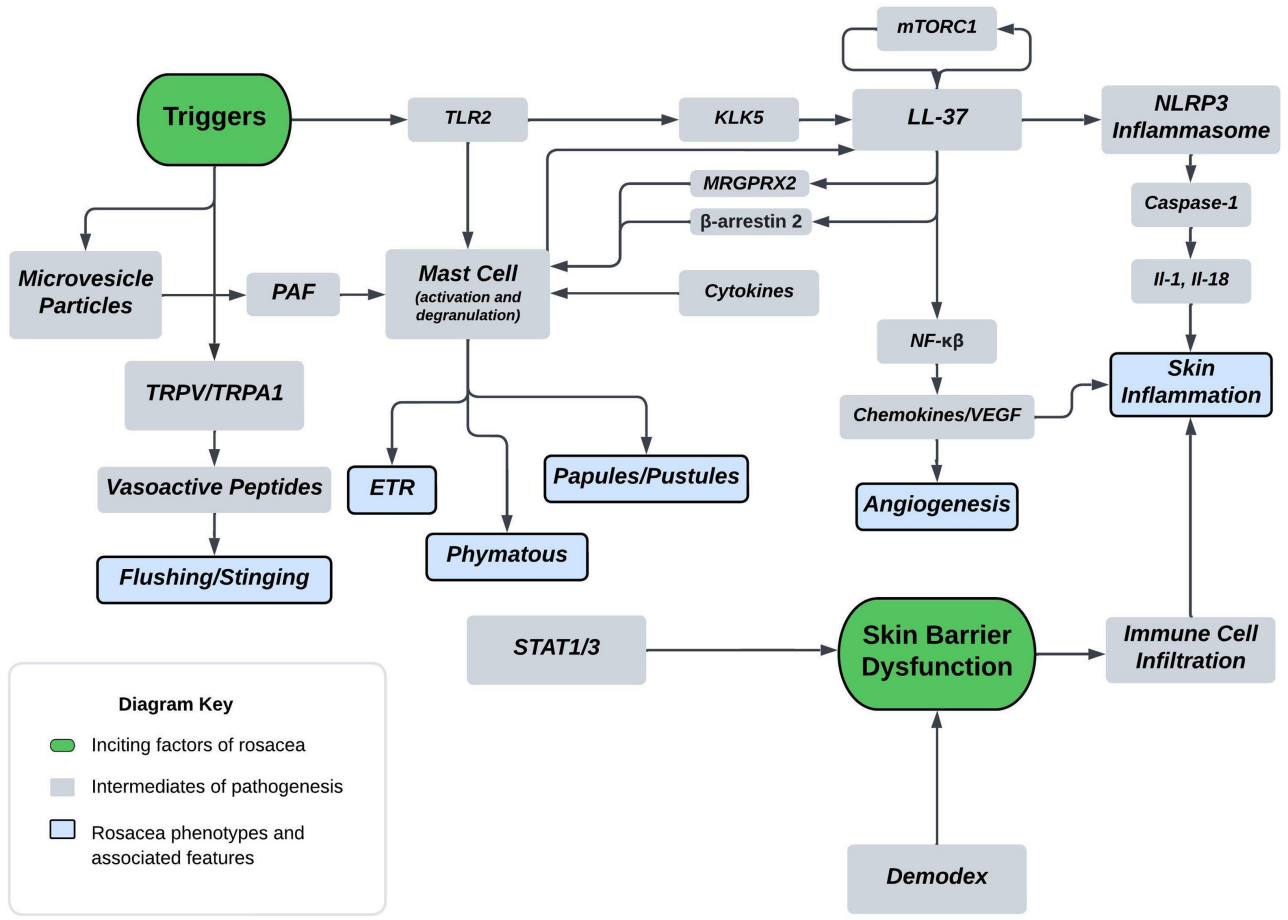
Proposed mechanism of rosacea pathogenesis. The flow diagram depicts inciting factors in green and molecular pathways in grey which ultimately leads to symptoms and rosacea phenotypes shown in blue. Some triggers of rosacea include PAMP’s, heat, and UVB. Major molecular players originating from triggers include TLR2, TRPV/TRPA, and MVP’s. TLR’s are sensors for PAMPs which act as triggers and induce KLK5 activation leading to the cleavage of cathelicidin LL-37 into pro-inflammatory fragments. Additionally, mTORC1 has been shown to interact with LL-37 in a feedback loop. Downstream effects of LL-37 fragments include mast cell activation, angiogenic chemokine release, and NLRP3 inflammasome activation. MRGPRX2 is a GPCR activated by LL-37, modulated by β-arrestin-2, and mediates non-IgE mast cell degranulation. Mast cell activation and degranulation is implicated in ETR, phymatous, and PPR rosacea phenotypes. Angiogenic chemokines include VEGF and lead to angiogenesis in the skin of rosacea patients. NLRP3 plays a pivotal role in innate and inflammatory signaling and functions through various cytokines, such as IL-1 β and IL-18. These cytokines play a pivotal role in skin inflammatory conditions with proven implications in PPR. Heat is another trigger, shown to act through the nociceptive TRPV/TRPA sensors leading to release of vasoactive peptides causing flushing and stinging. UVB is also a trigger that’s associated with MVP release from skin keratinocytes. MVP’s function through the pro-inflammatory mediator PAF causing mast cell degranulation. Skin barrier dysfunction is another inciting factor of rosacea and is associated with Demodex mite skin colonization and upregulation of STAT transcriptions factors. Skin barrier dysfunction leads to immune cell infiltration and skin inflammation in rosacea.

A thorough understanding of disease pathogenesis is important in determining rational therapeutic targets. As previously stated, the disease pathogenesis of rosacea is complex, multivariate, and not completely understood. However, known pathogenesis can fall into two, broad, overlapping domains: skin barrier dysfunction and environmental/genetic triggers. Figure 1 displays a flow diagram with the two discussed domains highlighted in green with phenotypic features highlighted in blue. The commonly associated pathways, cathelicidin family, transient receptor potential channels (TRP), mast cells, and NOD-, LRR-, pyrin domain-containing protein (NLRP3) inflammasome are shown in the diagram. The flow diagram is non-exhaustive but suffices to highlight pathogenesis in context of this review article. The following paragraphs provide a brief introduction of each pathway with relative implications in rosacea.

### 2.1 Skin barrier dysfunction

Researchers hypothesize that disrupted skin barrier promotes bacterial skin colonization and the combination of damaged and bacterial colonized skin, with their associated antimicrobial peptides (AMP’s), may trigger and activate rosacea (16). Quantitative measures of skin barrier dysfunction include transepidermal water loss (TEWL), decreased hydration, and elevated skin pH which all point to barrier dysfunction playing a role in rosacea pathogenesis (17). Medgyesi et al. studied skin barrier dynamics in PPR to assess the role of skin barrier alterations in rosacea. Overall, their results provide evidence that there are severe barrier alterations in facial skin of those with PPR. These findings have led the authors to suggest that skin barrier restoring therapies should be incorporated into rosacea management (18). Recently, signal transducer and activator of transcription factor 3 (STAT3) overexpression has been implicated in skin barrier dysfunction in rosacea. The upregulation and activation of STAT3 has known associations with psoriasis, however, understanding of its expression and role in rosacea remains limited. Wang et al. found that expression of STAT3 in keratinocytes of the epidermis was significantly increased in the rosacea patient compared to normal control using RNA sequencing. STAT3 contributed to skin barrier dysfunction patterns-related immune infiltration in rosacea (15). Overall, addressing skin barrier deficits appears integral in preventing and treating rosacea.

### 2.2 Cathelicidin

Cathelicidins are short cationic peptides known for their antimicrobial and immunomodulatory effects (19). In mouse models, long term exogenous cathelicidin LL-37 has been demonstrated to cause irreversible rosacea lesions, providing strong evidence for its role in rosacea pathogenesis (20). In humans, the cathelicidin protein LL-37 is implicated in the erythema and pustule phenotypes. This pathway is induced by toll-like receptors (TLRs) which are sensors for pathogen-associated molecular patterns (PAMPs) and danger-associated molecular patterns (DAMPs). Ultimately, elevated Toll-like Receptor 2 (TLR2) induces the serine protease Kallikrein-5 (KLK5) that cleaves LL-37 into pro-inflammatory fragments (11, 21, 22). Some downstream effects include mast cell activation, angiogenic chemokine release, and NLRP3 activation. MRGPRX2 is a G-protein coupled receptor (GPCR) on mast cells that mediates non-IgE mast cell degranulation. Recently, Probeski et al. has shown that this receptor is activated by LL-37 and may lead to aberrant activation of cutaneous mast cells in rosacea patients (23). Specifically, *in vitro* data by Yoon et al. (24) has shown that LL-37 promotes NLRP3-mediated inflammasome activation through multiple mechanisms ultimately leading to skin inflammation. Microbial infection, UV light, and injury are known stimuli that lead to mature LL-37 activation and its associated effects (25). Additionally, cathelicidin LL-37 has been shown to augment the UVB-induced IL-1β secretion by acting on the P2X7 keratinocyte receptor. Where the P2X7 receptor is located on multiple cells types and known to modulate many downstream effects, including inflammatory molecule release (26). Therefore, LL-37 modulates the proinflammatory and proangiogenic effects of UV radiation and contributes to sunlight sensitivity in rosacea (27). Another property of LL-37 is its ability to act as both a potent chemoattractant for mast cells as well as an augmenting agent to upregulate their antimicrobial properties (28, 29). Lastly, human Cathelicidin LL-37 has been shown to activate autophagy in human keratinocytes through mechanistic target of rapamycin (mTORC1) and MAPK pathways (30). Autophagy is an important process that functions to maintain cellular homeostasis in response to stressors, and autophagic keratinocyte dysregulation is associated with skin inflammatory conditions like rosacea and psoriasis (31, 32). Furthermore, hyperactivation of mTORC1 signaling has been found in the skin of rosacea patients, and ablation of mTORC1 signaling in mouse rosacea models blocks development of aggravated rosacea phenotypes (33). Overall, the cathelicidin LL-37 and its associated downstream effects are heavily implicated in rosacea pathogenesis.

### 2.3 Transient receptor potential channels

Transient Receptor Potential (TRP) channels are essential for modulating the driving force of ion entry across cell membranes. These channels include seven families implicated in a variety of diseases. Transient receptor pathway vanilloids (TRPV) are implicated in a variety of sensory neuropathies and are known to promote serotonergic and histaminergic itch responses (34). Upregulation of TRPV, specifically TRPV4 vasopeptide, has been implicated in flushing and stinging phenotypes of rosacea (35). Additionally, Transient Receptor Pathway Anykyrin (TRPA1) acts on nociceptive sensors and can potentiate inflammatory responses similar to that seen in rosacea (36). Lastly, Sulk et al. further proved the various TRPV’s role in ETR and PPR through dermal immunolabeling and quantifying gene expression. In ETR, they found dermal immunolabeling of TRPV2/TRPV3 and gene expression of TRPV1 significantly increased in rosacea skin biopsies. In PPR, the authors found enhanced dermal immunolabeling of TRPV3/TRPV4 and increased gene expression of TRPV3/TRPV1 (37). Overall, TRP vanilloid and anykyrin channels are implicated in both the ETR and PPR phenotypes of rosacea and are targets of therapy.

### 2.4 Mast cell activation and degranulation

The presence and activation of mast cells in an increased number has long been implicated in the pathogenesis of a variety of cutaneous disorders, including rosacea, atopic dermatitis, bullous pemphigoid, psoriasis, and many others (38). In rosacea, mast cells are known to contribute to the development and evolution of disease chronicity (39). Figure 1 displays some commonly associated pathways that can lead to mast cell dysregulation or activation and degranulation. Microvesicle particles (MVPs) are carrier molecules released from skin keratinocytes in response to stress and can carry pro-inflammatory molecules, like PAF, which has been shown to cause mast cell degranulation (40–42). Mast cells respond to TLR ligands by secreting cytokines, chemokines, and lipid mediators, and some studies have shown TLR ligand induces mast cell degranulation (43). Furthermore, Yamasaki et. all have shown a higher expression of TLR2 in patients with rosacea, which helps explain why these patients may have a higher inflammatory response to environmental stimuli (11). Additionally, a vast variety of cytokines and peptides are known to induce and be released from mast cells leading to characteristic histopathological features of the disease (44, 45). Ultimately, through a variety of mechanisms, mast cells appear to be an important effector cell type in rosacea pathophysiology and thus could play an important role in future therapeutics through new means (13).

### 2.5 NLRP3 inflammasome pathway

NLRP3 is an intracellular sensor that detects a wide range of factors including microbial motifs, endogenous danger signals, and environmental irritants leading to its formation (46). When formed, NLRP3 is a multiprotein complex, playing a pivotal role in innate and inflammatory signaling (47). It functions by activating pro-inflammatory cytokines IL-1β and IL-18. Aberrant inflammasome activation is associated with a wide variety of inflammatory disorders (48). Shown in Figure 1, LL-37, the cathelicidin protein, is implicated in various rosacea phenotypes through multiple mechanisms including the activation of the NLRP3 inflammasome pathway (24). Upon activation and formation of the inflammasome, the multi-protein complex promotes secretion of inflammatory cytokines IL-1β and IL-18 mediated by caspase-1 activation (49, 50). The IL-1 family of cytokines, which includes IL-1β and IL-18 are involved in a myriad of immune responses and implicated in multiple chronic inflammatory skin conditions (51). More specifically, the cytokine IL-1β has a variety of functions and end effects include enhanced expression of IL-8, TNF, and cyclooxygenase (COX-2), all of which are increased in rosacea, especially PPR (35, 52).

## 3 Current therapeutics

**Figure 2:**
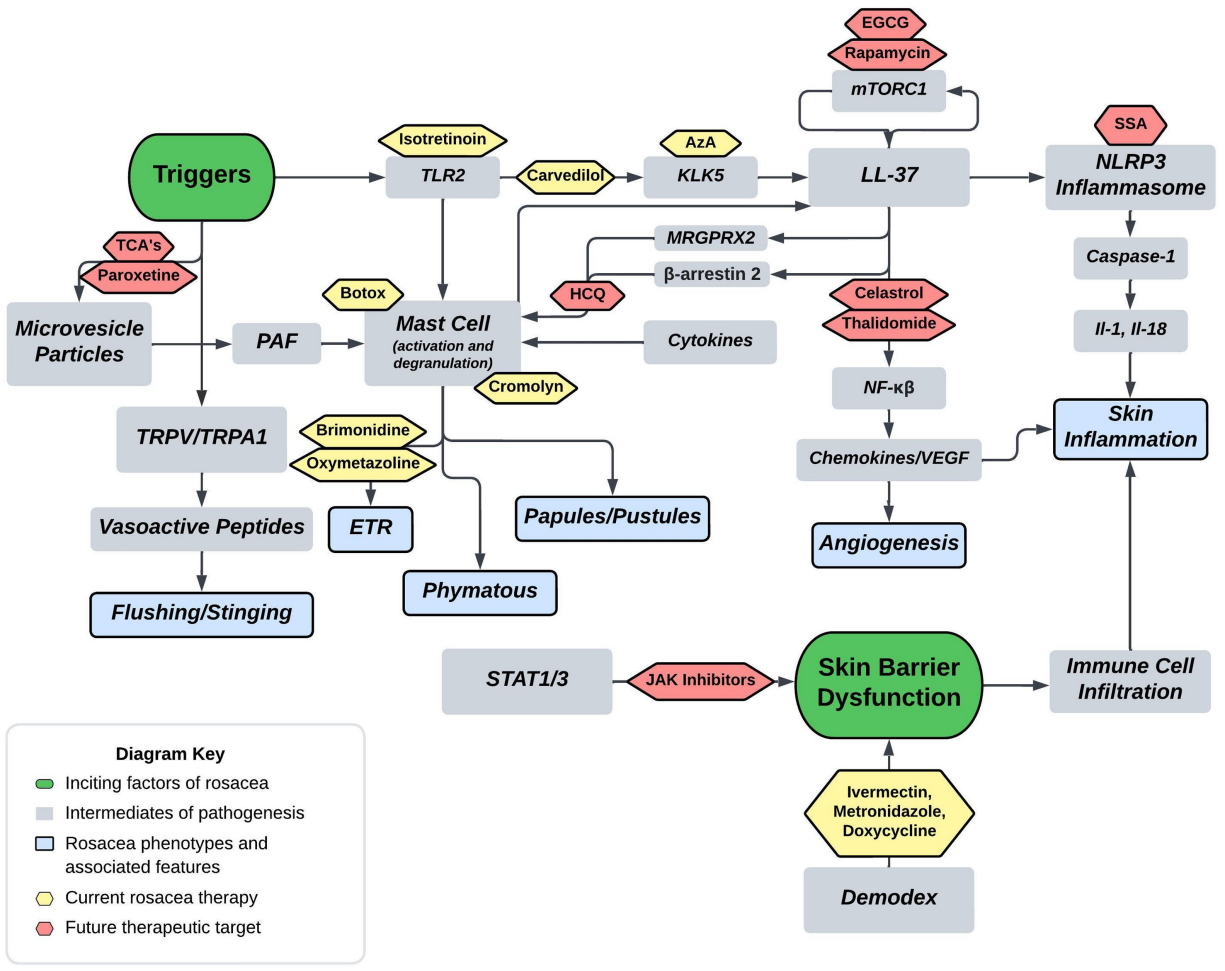
Proposed rosacea pathogenesis with potential drug targets. Current therapeutics are depicted in yellow with future therapeutic possibilities depicted in red. Aza, carvedilol, and isotretinoin are three current therapeutics that act on the cathelicidin pathway. Isotretinoin inhibits sebaceous gland function and downregulates the TLR2 pathway, carvedilol has been shown to downregulate TLR2/KLK5/LL-37 pathway, and Aza has been shown to downregulate KLK5. Botox and cromolyn are two current therapeutics that function via mast cell mediation. Botox has been shown to decrease mast cell degranulation in human and mouse models, and cromolyn functions as a mast cell stabilizer. Brimonidine and oxymetazoline are two current therapeutics that act as alpha-sympathetic receptor agonists in the treatment of ETR. Brimonidine acts via alpha-2 agonism and oxymetazoline acts via alpha-1 agonism. EGCG, rapamycin, celastrol, and thalidomide are four potential therapeutics that act on both downstream and upstream targets of LL-37. Rapamycin via mTORC1 modulation and EGCG has been shown to induce keratinocyte autophagy via a similar mTOR mediated pathway. Celastrol and thalidomide function by inhibiting the downstream effects of NF-kβ including release of various cytokines and chemokines. HCQ has been shown to suppress mast cell infiltration via hypothesized mitigation of LL-37 mediated activation through the GPCR MRGPRX2. SSA is hypothesized to treat rosacea via inhibition of NLRP3 inflammasome assembly. Lastly, TCA’s and paroxetine are hypothesized to block the release of MVP’s from keratinocytes thereby mitigating mast cell degranulation from PAF (Tricyclics = tricyclic antidepressants, HCQ = hydroxychloroquine, EGCG = epigallocatechin-3-gallate, SSA = supramolecular salicylic acid, AzA = azelaic acid).

Current FDA approved therapies include topical and oral pharmacotherapy, laser and light therapy, and other surgical procedures. Laser and light therapy modalities and surgical procedures are beyond the scope of this review. Optimal medical management of rosacea involves evaluating each patient’s unique presentation to customize multiple modalities of therapy (53, 54). For each medication, Figure 2 depicts the method of action in relation to the pathophysiology shown in Figure 1. Table 1 provides additional summary of each medication category, the proposed method of action, and the targeted rosacea phenotype.

**Table 1:** Current recommended treatment methods.

Category	Treatment	Proposed MOA	Targeted phenotype(s)
General measures	Cleansers, UV protection, and moisturizers	Multi-variate	All
Topical	Brimonidine	Alpha-2 agonist	Flushing and erythema
	Oxymetazoline	Alpha-1 agonist	Flushing and erythema
	Ivermectin	Anti-inflammatory	Papules and pustules
	Azelaic acid	KLK-5 inhibition	Papules and pustules
	Sodium sulfacetamide	Anti-inflammatory	Papules and pustules
	Metronidazole	Anti-microbial and anti-inflammatory	Papules and pustules
	Benzoyl peroxide	Anti-microbial and anti-inflammatory	Papules and pustules
Oral	Doxycycline	Anti-microbial and anti-inflammatory	Papules and pustules
	Minocycline	Anti-microbial and anti-inflammatory	Papules and pustules
Off-label	Isotretinoin	Inhibition of sebaceous gland function, TLR-2 downregulation	Papules and pustules
	Carvedilol	Non-selective β and α-1 antagonist, cathelicidin downregulation	Flushing and erythema
	Cromolyn	Mast cell stabilization	Flushing and erythema
	Botulinum toxin	Acetylcholine blockade, decrease mast cell degranulation, anti-inflammatory	All

Each treatment is categorized into general measures, topical, oral, or off-label. Each treatment method is a presented with a proposed method of action (MOA) and targeted phenotype(s) for treatment.

### 3.1 General measures

Skin barrier and permeability dysfunction plays a significant role in rosacea pathogenesis and severity, therefore various treatment methodologies aim to restore the skins native protection capabilities and are viewed as general measures in rosacea therapy. The basic features of skin barrier treatment include cleansers, UV radiation protection, and moisturizers (17). Improvement of barrier dysfunction both helps reduce inflammation and decreases the interaction of irritating particles (55). Skin cleansers should aim to remove pollutants and debris while maintaining physiologic pH as cleansers with elevated pH may damage the stratum corneum or destroy essential components of the skin (17, 56). UV light is implicated in all rosacea presentations and is known to increase inflammation, angiogenesis, telangiectasia, and fibrosis, which warrants proper photoprotection with sunscreen and avoidance of UV radiation (57). Moisturizers for barrier maintenance should include ingredients such as emollients, humectants, and as tolerated, occlusives (58). Previous studies have shown the efficacy of sunscreen and moisturization in increasing electrical capacitance and decreasing TEWL, two measures of skin barrier dysfunction (59). Overall, cleansers, moisturizers, sunscreen, and UV avoidance play an integral role in the treatment of rosacea.

### 3.2 Topical agents

There are nine FDA-approved agents for rosacea (60). For flushing and erythema, guidelines state that two approved topical therapeutics can be used (61). Brimonidine is an alpha-2 agonist known to reduce inflammation and edema in these phenotypes. Oxymetazoline, an alpha-1 agonist is also indicated for reducing persistent facial erythema with or without papulopustular lesions (62). For papules and pustules topical medications such as ivermectin, azelaic acid, sodium sulfacetamide, metronidazole and encapsulated benzoyl peroxide may all be utilized (60, 61, 63). Topical ivermectin (IVM) has been associated with anti-inflammatory effects but correlation of effects with rosacea are unknown. Recent studies reported downregulation of LL-37, IL-8, TLR-4, and HBD-3 following 12 weeks of topical IVM use in rosacea patients. Additionally, the role of IVM in reduction of Demodex mite is a current targeted area of research (64). Next, azelaic acid (AzA) was one of the first 3 FDA-approved drugs specific for the treatment of rosacea and acts to downregulate the cathelicidin pathway via inhibition of kallikrein-5 (65, 66). A recent clinical trial combined topical AzA with dihydroavenanthramide to reduce negative side effects of AzA (67). Sulfur sulfacetamide is effective for mitigating erythema and inflammation through an unknown anti-inflammatory mechanism, and can be used in combination with other agents for treatment (68, 69). The antibiotic metronidazole has been approved for the treatment of rosacea since 1990, and functions as an anti-microbial and anti-inflammatory agent that helps maintain the skin barrier (70, 71). Encapsulated benzoyl peroxide (BPO) was FDA-approved for treatment of rosacea in April, 2022. BPO has previously been too irritating for skin of those with rosacea, but through administration in an encapsulated vehicle, the medication can slowly be delivered through the skin (72).

### 3.3 Oral agents

Oral medications for rosacea include tetracyclines, like doxycycline and minocycline, which are both indicated for the PPR phenotype (61). Tetracyclines are commonly prescribed antibiotics that also exhibit anti-inflammatory activities at sub-antimicrobial doses (73). Sub-antimicrobial doses allow for reduction of inflammatory lesions with less incidence of antibiotic resistance (74). Additionally, tetracyclines have been shown to regulate the LL-37 pathway, inhibit matrix metalloproteinases, and reduce inflammation (62). A recent systemic meta-analysis found minocycline as the most effective and safe antibiotic therapy for reducing papules and pustules in PPR (75). Overall, oral tetracyclines play an integral role in rosacea therapy.

### 3.4 Off-label agents

Off label therapeutics for rosacea therapy may include isotretinoin, carvedilol, cromolyn, and botulinum toxin (BTX). Isotretinoin is a retinoid derivative primarily utilized for refractory acne vulgaris but also shows efficacy in management of refractory rosacea (76). The drug’s primary function is inhibition of sebaceous gland function but has also been shown to downregulate the TLR-2 pathway (77, 78). Carvedilol is another off-label medication used in rosacea to reduce persistent facial flushing and erythema (79). It is a non-selective beta blocker, with alpha-1 adrenergic antagonistic activity, that also functions as a potent antioxidant (80). Carvedilol was shown by Zhang et al. to downregulate the TLR2/KLK5/cathelicidin pathway. TLR2, KLK5, cathelicidin peptides, and CD68 are all highly expressed in patients with rosacea and rosacea animal models. Carvedilol has been recommended since 2011 for flushing symptoms in refractory cases of rosacea (81). Additionally, cromolyn, a known mast cell stabilizer is a treatment option for reducing redness and flushing (82, 83). Botulinum Toxin (BTX) is another treatment option in rosacea for reducing flushing and inflammation through mechanisms such as, blocking acetylcholine receptors from peripheral autonomic nerves, and release inhibition of inflammatory mediators like substance P (84). Also, BTX has been shown to significantly decrease mast cell degranulation in both human and mouse mast cells (85). Overall, off-label medications including adrenergic mediators, mast cell stabilizers, and acetylcholine mediators, have proven efficacy in rosacea management.

## 4 Future therapies and rational targets

Research into the pathogenesis of rosacea is trending upward owing to rapid discoveries in the field, which indicates pathophysiology has attracted attention for future research (86). Novel discoveries increase our understanding of intrinsic and extrinsic pathways contributing to rosacea and allow for new opportunities of therapeutics. Descriptions of future therapies are broken down into subsections of implicated pathogenesis such as skin barrier dysfunction, cathelicidin pathway, mast cell targets, and microvesicle particles. Each pathogenic target then discusses proposed medications, and hypothetical targets based upon pathogenic insight. For reference, Figure 2 depicts the future treatment targets in relation to pathophysiology and Table 2 provides a summary of each target, proposed mechanism of action, and example medications.

**Table 2:** Future therapies and rational targets.

Therapeutic target	Mechanism of action	Example medication(s)
Skin barrier	JAK/STAT therapy	Tofacitinib
Cathelicidin pathway	mTOR regulation	Rapamycin, celastrol
	Keratinocyte autophagy induction	EGCG
	Cytokine/chemokine reduction	Thalidomide
	NF-kβ suppression and NLRP3 inflammasome inhibition	SSA
	TLR modulation	NC
Mast cell	LL-37 mediated mast cell activation	Hydroxychloroquine, artemisinin
	β – Arrestin modulation	NC
MVP	aSMAse inhibition	TCA’s, paroxetine

Each mechanism of action and example medication(s) is categorized by either skin barrier, cathelicidin pathway, mast cell, or microvesicle particle (MVP) targets. NC = None current, meaning there is no current example medication acting on the pathway or there are potential medications in various stages of drug discovery.

### 4.1 Skin barrier dysfunction targeted therapy

Rosacea is associated with a profoundly diminished skin barrier and prominently impaired permeability that promotes bacterial colonization leading to rosacea phenotypes (18). Known mechanisms of addressing barrier deficiency includes use of cleanser and moisturizer formulations that restore skin hydration, normalize pH, and restore the skin microbiome (87). Recently, signal transducer and activator of transcription factor 3 (STAT3) overexpression has been implicated in skin barrier dysfunction in Rosacea (15). The upregulation and activation of STAT3 has known associations with psoriasis, however, understanding of its expression and role in rosacea remains limited. Wang et al. found that expression of STAT3 in the keratinocyte of the epidermis was significantly increased in rosacea patients compared to normal control using RNA seq. STAT3 contributed to skin barrier dysfunction patterns-related immune infiltration in rosacea (15). Recently, STAT1 was discovered to mediate keratinocyte-immune cell crosstalk in the skin with major implications in rosacea pathogenesis as it relates to skin barrier and immune cell activation (88). Given the upregulation of STAT transcription factors, inhibitors of these pathways may serve as potent therapeutics. Janus kinase (JAK) and STAT (JAK/STAT) signaling plays an important role in keratinocyte associated skin disease and pathway inhibitors show efficacy in other disease such as psoriasis and atopic dermatitis (89). In a previous rosacea clinical trial, the JAK inhibitor tofacitinib was shown to significantly ameliorate erythema in 72.4% of enrolled patients (90). The efficacy of JAK/STAT inhibitors should theoretically be tied to mitigation of skin barrier deficiencies. Ultimately, skin barrier dysfunction is implicated in all phenotypes of rosacea and future therapies should target new pathogenic insights such as the roles of STAT1 and STAT3.

### 4.2 Cathelicidin pathway

As discussed previously, cathelicidin LL-37 is an anti-microbial effector molecule of the innate immune system, and in rosacea, exhibits defects in expression, function and processing (91). Figure 1 highlights the LL-37’s role in rosacea pathogenesis, including its actions on mast cells, NF-kβ activation, and promotion of NLRP3 mediated inflammasome activation. Additionally, Figure 1 depicts that TLR-2 responds to PAMPs and DAMPs which induces KLK-5 to cleave and activate LL-37. Mammalian target of rapamycin pathway, mTORC1 has been shown to regulate the cathelicidin through a feedback loop, and hyperactivation of its signaling aggravates rosacea features and is required for angiogenesis in the development of rosacea (33, 92). Rapamycin (Sirolimus) is a modulator of mTORC1, that is under investigation as a potential therapeutic. Topical rapamycin has been shown to significantly improve rosacea symptoms through its antiangiogenic and antiproliferative properties (33, 93). The mTOR pathway is a regulator of autophagy in keratinocytes, and reduced autophagy was shown in keratinocytes of a rosacea mouse model (31, 94). Importantly, it has been described that autophagy protects keratinocytes against injury in inflammatory diseases (95). Epigallocatechin-3-gallate (EGCG) is a natural polyphenol identified as a potential therapeutic in various skin inflammatory conditions and ultimately shown by Zhou et al. to reduce rosacea-like inflammation by inducing keratinocyte autophagy (31). Celastrol, is a plant derived triterpene carrying anti-inflammatory and anti-oxidant activities that may serve as another potential drug for regulating inflammation and angiogenesis (96). The drug functions by inhibiting the Ca^2+^-CaMKII-dependent mTOR-NF-kβ signaling pathway (97). Continuing the theme of LL-37 modulation for treatment of rosacea, thalidomide has been shown to alleviate rosacea phenotypes in a mouse model by reducing the production of cytokines and chemokines induced through LL-37 (98, 99). Another medication with therapeutic potential around the cathelicidin pathway is supramolecular salicylic acid (SSA). Studies have demonstrated that SSA displays keratolytic, antibacterial, and anti-inflammatory properties and is currently under research for efficacy in rosacea (100). The therapy is hypothesized to take effect through suppression NF-kβ and inhibition of assembly of the NLRP3 inflammasome (24, 101). Toll-like receptors, specifically TLR-2, are implicated in the overactivity in the cathelicidin pathway through their response to PAMPs and DAMPs. Due to their increased expression in those with rosacea, TLR2 may serve as a molecular target for future therapeutics. TLR antagonists are currently being investigated for treatment of inflammatory diseases, specifically TLR7/TLR9 in systemic Lupus Erythematosus, and TLR4 in sepsis and allergies (102–104). Through drug discovery methods, novel medications such as NPT1220-312, a TLR2/TLR9 antagonists, are being analyzed for their anti-inflammatory properties through mediation of the TLR2 primed NLRP3 inflammasome and cytokine/chemokine release (105). Overall, cathelicidin LL-37 and its associated effectors play a major role in rosacea pathogenesis and future therapeutics will inevitably target players in its pathway such as mTORC1 and TLR2.

### 4.3 Mast cell pathway targeted therapy

Recently, researchers have provided compelling data suggesting that mast cells serve as key players in the pathogenesis of rosacea, especially as a source of LL-37 and KLK5 (83, 106–108). Due to the integral role mast cells play in pathogenesis, interventions targeting their activation, degranulation, and downstream effects could mitigate symptoms of rosacea. The anti-malarial drug, hydroxychloroquine, was recently shown in experimental murine rosacea models to suppress mast cell infiltration through mitigation of LL-37 mediated activation (109). Recent human clinical trials indicate hydroxychloroquine can produce improvement in rosacea (110). Artemisinin is another anti-malaria drug with proven efficacy for rosacea in mouse models, hypothesized to act through a similar mechanism of mast cell mediation as hydroxychloroquine (83, 111). A recent discovery in mast cell activation is the role MRGPRX2 in rosacea mouse models. MRGPRX2 is a GPCR expressed by mast cells, implicated in non-IgE mediated mast cell degranulation, and has been shown to be activated by LL-37 (23). Aberrant activation of cutaneous mast cells may be involved through this mechanism (112). Release of chemokines by neuropeptides via MRGPRX2 promotes neurogenic inflammation and mast cell activation in rosacea. β-arrestin-2 modulates MRGPRX2 by promoting cofilin dephosphorylation, ERK 1/2 and NF-kB phosphorylation, mast cell chemotaxis, and chemokine/cytokine generation (113). This discovery of a GPCR newly implicated in rosacea may serve as a drug target. β-arrestins modulate many physiological processes and their upregulation are associated with a variety of diseases. There are huge therapeutic potentials for β-arrestin modulation to serve as disease specific therapies, and in rosacea a modulator could hypothetically reduce aberrant mast cell activation through the previously described mechanism (114).

### 4.4 Microvesicle particles targeted therapy

Microparticles are membrane vesicles derived from cells undergoing stress and have been shown to mediate cell communications by transferring particles like membrane proteins, phospholipids, and RNAs form parent cells (115). UVB is a stressor from sunlight exposure that damages DNA and has toxic effects on multiple cell types, including keratinocytes (116). Bihl et al. have shown that a UVB damaged keratinocyte cell line generates microvesicle particles (MVP), with platelet activating factor (PAF) playing a major signaling role in the process (117). MVPs have been proven to cause local and systemic effects and are implicated in erythema. Of note, downstream effects of MVP’s include the ability to transport protein cytokines. In addition, recent studies indicate that MVP can transport the glycerophosphocholine-derived lipid mediator PAF, a pro-inflammatory lipid mediator known to cause downstream effects such as mast cell degranulation and the wheal and flare response seen in allergic reactions (40, 41). Topical acid sphingomyelinase (aSMAse) inhibitors include topical agents like tricyclic antidepressants and have been shown to mediate MVP production by blocking their release from keratinocytes (42, 118, 119). Additionally, the SSRI paroxetine has displayed proven effectiveness in one randomized control trial for the treatment of refractory erythema in rosacea patients (119, 120). Although its mechanism of action is assumed in relation to serotonin modulation mediated vasoregulation, paroxetine has proven aSMAse activity and therefore it can be hypothesized to modulate MVP activity as well. Blockage and inhibition of MVP release form keratinocytes may mitigate erythema in rosacea. Overall, aSMAse is an important mediator enzyme of MVP release form keratinocytes. TCA’s such as imipramine and amitriptyline, and the SSRI paroxetine, been shown to inhibit aSMAse which normally catalyzes the hydrolysis of sphingomyelin to ceramide (121). Imipramine and amitriptyline have been previously shown to block the ability of multiple agents to trigger MVP production from keratinocytes and skin (42, 118). Future trials will look for the efficacy of imipramine, amitriptyline and other aSMAse inhibitors in mitigating erythema in rosacea. Moreover, MVP and other subcellular particles released from skin could provide a mechanism for extracutaneous manifestations for rosacea such as headaches.

## 5 Conclusion

As previously discussed, rosacea is a chronic disease with well-documented morbidity and mortality. However, health insurance companies view this condition as increasingly cosmetic, and many drugs are not covered under formulary guidance. Patients whose condition is refractory to doxycycline or metronidazole may only be able to consider other treatments if they can afford non-coverage prices. Additionally, the tetracyclines currently covered by insurance are usually a generic 50 – 100 mg doxycycline or minocycline formulation rather than the newer, low-dose sustained formulations. For example, Aetna’s medical clinical policy bulletin for rosacea therapy deems topical minocycline and botulinum toxin under research and investigational although there has been proven efficacy. With these considerations, this may open the door for cheaper, repurposed therapeutics, such as topical TCA or SSRI mediated MVP modulation discussed in this review, if proven efficacy is shown.

There is an increasing trend into research and discovery of rosacea’s pathogenic process. As discoveries into the mechanisms are published, the amount of prospective therapeutics increases. This review paper sought to highlight new therapeutic drugs in various phases of discovery and new targets based upon pathogenic insights. Maintenance of a thorough skin barrier is integral in treating rosacea, and current treatment mechanisms may be supplemented with JAK tyrosine kinase inhibitors. Targeting the cathelicidin pathway and its downstream effectors is a major source of current therapeutics and a major target for future treatments. Mast cell activation and degranulation is another pathogenic target of many medications, and future therapeutics may utilize β-arrestin modulation. Lastly, ongoing studies have implicated MVP release as potential effectors in local and systemic inflammatory processes and topical TCA’s or SSRI’s may moderate these effects. Overall, recent studies have provided important insights and targets in rosacea. As further pathogenic insights are developed more effective therapies will become available.

## Author contributions

GF: Writing – original draft, Writing – review & editing. JT: Methodology, Supervision, Writing – review & editing. CR: Resources, Supervision, Writing – review & editing.
